# Aetiology of idiopathic scoliosis: the “scotch type“ effect or the abnormal initial local anterior-lateral conjunction between the dura mater spinalis and the periosteum of spinal canal of concave side. New evidence

**DOI:** 10.1186/1748-7161-9-S1-O19

**Published:** 2014-12-04

**Authors:** Valerii Drobyshevskiy

**Affiliations:** 1 G.I.Turner Scientific and Research Institute for Children’s Orthopaedics, St-Petersburg, Russian Federation

## Background information

Early we described a local internal lateral fixation (LILF) of a dura mater spinalis to the wall of the vertebral channel as the main reason of the serious idiopathic scoliosis.

There are a postmortal investigations of the serious scoliosis with a LILF of duramater. Early it was considered by other author that this a secondary phenomenon as a consequence of the vertebras edges pressure in the vertebral channel towards the dura mater.(Movshovich I.,1964 [1]).

## Purpose

First , to prove that the LILF is not the secondary phenomenon, on the contrary , the LILF of dura mater like a hooked bowstring causes a serious idiopathic scoliosis.

Second, to find a trace on MRI of flat tension of dura mater in consequence of conjunction between the Dura matter and the Periosteum of spinal canal of concave side.

## Method

Now we analyzed the evolution of the vertebral foramen forms on 10 postmortal cases of the serious idiopathic scoliosis and 30 MRI-tests of the initial little idiopahtic scoliosis with 'bad scenario" or the intramedullar cyst.

We used the Cheneau - Abbott type braces with the non-magnetic parts and the side position of patient for MRI-test. We investigated the form and the locations of the spinal cord in the corrected position of the scoliosis spine.

## Results

We found trace of flat tension of dura mater in consequence of conjunction between the Dura matter and the Periosteum of spinal canal of concave side.

We find that the starting scoliosis have LILF **of** dura mater in several cases. These are serious scoliosis in future.

## Conclusions and discussion

The MRI test for a LILF of the dura mater can help to make a forecast of future development scoliosis. We can make the early scoliosis treatment by separation of the LILF of the dura mater like Edville Gerhardt Abbott (1913) [2] by overcorrection brace. Now it is called the Abbott-Cheneau brace.
